# Traumatic renal injuries: the prevalence, management and outcome of renal vascular injury

**DOI:** 10.1007/s00068-026-03389-w

**Published:** 2026-11-12

**Authors:** Seppo K. Koskinen, Sune Sun, Zlatan Alagic, Anders Enocson, Anna Kistner

**Affiliations:** 1 Department of Diagnostic Radiology, Karolinska University Hospital, Stockholm, 171 76 Sweden; 2 Department of Clinical Science, Intervention, and Technology, Division for Radiology, Karolinska Institute, Stockholm, 171 77 Sweden; 3 Department of Molecular Medicine and Surgery, Karolinska Institute, Stockholm, 171 77 Sweden; 4 Department of Trauma, Acute surgery and Orthopaedics, Karolinska University Hospital, Stockholm, 171 76 Sweden; 5 Department of Nuclear medicine and Medical Physics, Karolinska University Hospital, Stockholm, 171 76 Sweden

**Keywords:** Renal trauma, Renal vascular injury, Computed tomography, Conservative treatment, Angioembolization

## Abstract

**Purpose:**

To assess the prevalence, management and outcome of renal vascular injuries (RVIs; active bleeding and contained vascular injuries) seen on admission CT. Also, we wanted to find out the prevalence and survival of patients with concomitant injury to liver, spleen, and kidney seen on admission CT.

**Methods:**

We conducted a retrospective, single-center, longitudinal cohort study with nine-year data (2013–2021) of all ≥ 15-year-old patients with renal injury identified in a trauma registry. Admission CT and angiographic examinations were reviewed to assess RVIs, including active bleeding, arterial dissection, segmental infarct, pseudoaneurysm or AV-fistulae. Medical records were reviewed for treatment, hospital length of stay and 30-day survival rate.

**Results:**

Out of 2805 patients with abdominal trauma, 243 (8.7%) had a renal injury, of whom 182 (74.9%) underwent CT on admission. RVI was found in 56 patients (30.8%). Among these 56 patients, 42 (75%) had active bleeding, eight (14.3%) segmental infarct, six (10.7%) pseudoaneurysm, and three (5.4%) arterial dissection. Of these, ten patients (17.9%) underwent angioembolization, and three (5.4%) underwent laparotomy. Patients with RVI were slightly older than those without RVI (43.6 vs. 37.8 years, *p* = 0.0409) and had more often a penetrating trauma (OR 2.836; 95% CI 1.127–7.136, *p* = 0.0416). Hospital length of stay (14.7 vs. 14.9 days, *p* = 0.9520) and 30-day survival (95.2% vs. 92.6%, *p* = 0.3165) did not differ significantly between patients without and with RVI. Among patients with available CT, fourteen (14/182; 7.7%) had concomitant injuries to the liver, spleen and kidney, with no statistically significant difference in 30-day survival compared with patients without such concomitant injuries (92.9% vs. 94.6%; *p* = 0.5237).

**Conclusion:**

RVI was not uncommon in our study cohort, occurring in 30.8% of patients with renal injury who underwent CT on admission. Of these, 17.9% were treated with angioembolization. Patients with RVI were slightly older and were more likely to have a penetrating injury than patients without RVI. No statistically significant difference in hospital length of stay or 30-day survival was found between patients without and with RVI.

## Introduction

Renal injury is seen in 1–5% of all trauma patients and is most often due to blunt trauma [1, 2]. Penetrating injuries, though less common, are associated with significantly higher rates of vascular involvement and operative intervention [3]. Renal vascular injury (RVI), i.e. injuries to the main renal vessels, and their segmental branches, represents the most severe subset with reported incidences of 3–15% among all renal trauma cases [4, 5]. Injuries to the renal hilum are life-threatening emergencies that demand rapid diagnosis and intervention, as delayed recognition may have catastrophic and irreversible consequences. Renal vein injuries, on the other hand, carry a substantial hemorrhagic risk due to the high-flow and low-resistance nature of the venous system [6].

Multi-detector CT (MDCT) with intravenous contrast is currently the imaging method of choice in polytrauma [7, 8]. MDCT angiography (MDCTA) in particular, has transformed the diagnostic landscape for renal vascular trauma and enables rapid, non-invasive diagnosis of renal vascular injuries [2].

The main purpose of this study was to assess the prevalence, management and outcome of RVIs (active bleeding and contained vascular injuries, such as pseudoaneurysm/AV-fistula) seen on admission CT in patients sustaining traumatic renal injuries. Also, as a substantial number of patients with high-grade renal injury have injuries to other abdominal organs [1], we aimed to investigate the prevalence and survival of patients with concomitant injuries to the three highly vascularized and most frequently injured abdominal organs: the liver, spleen, and kidneys.

## Materials and methods

**Fig. 1:**
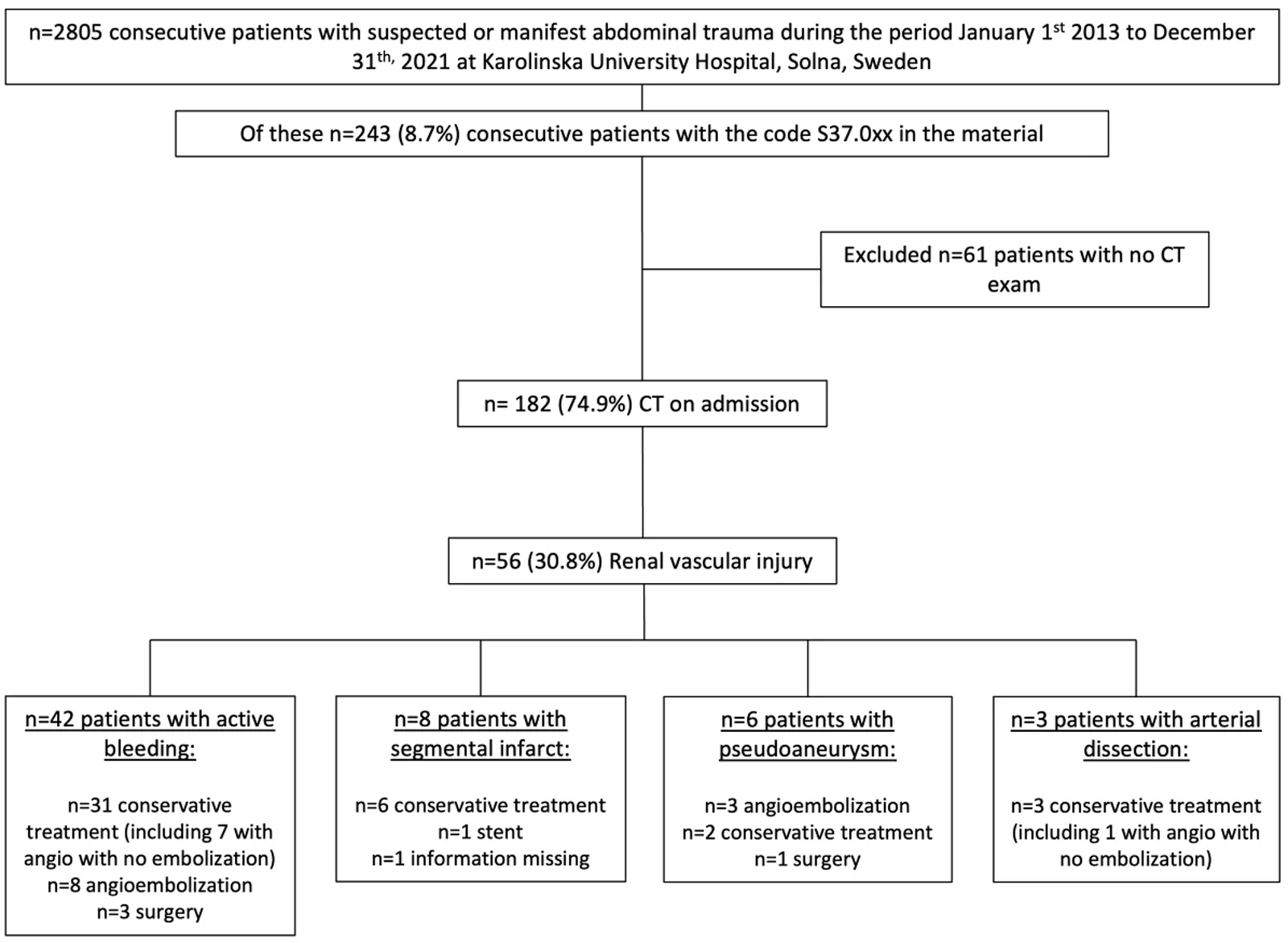
Renal vascular injuries in patients with abdominal trauma at Karolinska University Hospital between 2013–2021

Approval from the local ethics committee was obtained for this retrospective, longitudinal cohort study (Ethical application number 2019–05643, 2022-02753-02) and therefore informed consent was waived. The collection of patients and data has been previously described in our earlier publication [9]. In brief, nine-year data (2013–2021) of all patients with severe abdominal trauma were retrieved from the local trauma register of a level 1 trauma center (Fig. 1).

The trauma team consisted of an anesthesiologist, a general surgeon, an orthopedic surgeon, a radiologist, and nursing staff.

Patients aged 15-years and older with an ICD code for renal injury (S37.0XX) were included. Patients without an adequate admission CT were excluded from the analysis of renal vascular injuries. The following parameters were extracted from the trauma register and/or electronic medical records: age, gender, Injury Severity Score (ISS), New Injury Severity Score (NISS), 30-day mortality, injury mechanism, surgical and/or radiological interventions (angioembolization), and hospital length of stay (LOS). The CT images were retrieved from the local picture archiving and communication systems (the radiological information system (RIS)/picture archiving and communication system (PACS); SECTRA AB, Linköping, Sweden), and the CT protocol was recorded.

CT and angiographic examinations from all eligible patients were identified and the reports and images were reviewed by SS, a radiologist with 4 years of experience. Equivocal cases were resolved by consensus with SS. All CT examinations had previously been interpreted clinically by at least one radiology resident and one senior radiologist. The final study group comprised patients with an adequate admission CT and a renal injury verified by CT or surgery.

The renal injuries seen on CT were graded according to the AAST Injury Grading Scale 2018 revision. A vascular injury was defined as segmental infarct, arterial dissection, active bleeding (contrast extravasation), or a contained vascular injury (pseudoaneurysm or arteriovenous fistula), i.e. a focal collection of vascular contrast that decreases in attenuation with delayed imaging [10, 11].

Treatment data were extracted from electronic medical records and, when applicable, from angioembolization data available in PACS.

### CT technique

The multitrauma CT examinations at our hospital were performed on either a 256-slice multi-detector CT (MDCT) scanner (Revolution CT (RevCT), GE Healthcare, Milwaukee, Wisconsin, USA) or a 64-slice MDCT scanner (LightSpeed VCT (VCT), GE Healthcare, Milwaukee, Wisconsin, USA). In 2015, the RevCT was installed in the trauma department and replaced the VCT. Concurrently, the standard single-phase venous trauma CT protocol was revised for high-level trauma patients to include an additional whole-body arterial phase, resulting in a multi-phase protocol. Low-energy trauma patients continued to undergo single-phase venous trauma CT on the RevCT. On the RevCT, the multi-phase trauma CT protocol was performed with tube currents of 150 mA in the arterial phase and 120 mA in the venous phase, with tube voltages of 100 kV and 120 kV, respectively. For obese patients the tube current in the arterial phase was 300 mA and in the venous phase 255 mA and the tube voltage was 120 kV for both phases. In the single-phase trauma CT protocol on the RevCT, the used tube current was 225 mA and the tube voltage 120 kV, and for obese patients 300 mA and 120 kV. In the single-phase trauma CT protocol on the VCT, the used tube current was 150–200 mA and the tube voltage 120 kV. For the multi-phase trauma CT protocol performed on the RevCT, SmartPrep™ (Ge Healthcare) was used for bolus triggering of the arterial phase, followed by venous-phase imaging 45.0 s after the arterial acquisition ended. In single-phase protocols, the scan delay was 65.0 s on the RevCT and 55.0–60.0 s on the VCT. When clinically requested, delayed-phase images were acquired approximately 8 min after the venous-phase acquisition. The rotation time was 0.5 s for all protocols and enhancement phases on the RevCT, and 0.4 s on the VCT. The pitch was 0.992 for all phases on the RevCT, and 0.984 on the VCT. The detector coverage was 80 mm for all phases on the RevCT, and 40 mm on the VCT. Either ioversol or iohexol, both containing 350 mg iodine/ml, were administered as intravenous contrast medium. Flow rates were set to approximately 4.5-5 ml/s when an arterial phase was included and to 2.5–3.5 ml/s for venous phase-only scans. The contrast dose was calculated as 500 mg iodine/kg, with maximum body weights for dose calculation set at 80 kg for women and 100 kg for men.

### Statistics

The Mann-Whitney U test was used for the comparison of ordinal data, and the unpaired t-test for continuous data. The chi-square and Fisher’s exact tests were used to compare categorical data. Multiple comparisons were done with ANOVA and Kruskal-Wallis test for continuous and ordinal data, respectively. For pairwise comparisons, Dunn’s test was used. Odds ratios were calculated to analyze survival rates and difference in penetrating vs. blunt trauma injury mechanism. Statistical analyses were performed using a commercial software package SAS/STAT v.9.4 (SAS Institute Inc., Cary, NC, USA).

## Results

Patient characteristics are summarized in Tables 1 and 2.

**Table 1:** Patient characteristics; kidney injuries

Characteristics	
No. of patients (n)	243
Age (years)	38.5 (15–83)
Sex	
Male	192(79.0.%)
Female	51 (21.0%)
ISS	26 (4–75)
ISS > 15 (n)	203 (83.5%)
NISS	34 (4–75)
NISS > 15 (n)	216 (88.9%)
LOS days	14.5 (1-180)
Survived to discharge (reciprocal of 30-day mortality)*	201 (83.7%)

Values are given in mean (age, LOS) or median (ISS, NISS). Range in parentheses

ISS, Injury Severity Score; NISS, New Injury Severity Score; LOS, hospital length of stay

*In three cases this information was missing

**Table 2:** Patient characteristics; kidney injuries, CT available

Characteristics	
No. of patients (n)	182
Age (years)	39.6 (15–83)
Gender	
Male	146 (80.2.%)
Female	36 (19.8%)
ISS	22 (4–75)
ISS > 15 (n)	144 (79.1%)
NISS	27 (4–75)
NISS > 15 (n)	157 (86.3%)
LOS days	14.7 (1-180)
Survived to discharge (reciprocal of 30-day mortality)*	172 (95.0%)

Values are given in mean (age, LOS), numbers (gender, survived to discharge) or median (ISS, NISS). In parentheses range or for gender/survived to discharge percentage

ISS, Injury Severity Score; NISS, New Injury Severity Score; LOS, hospital length of stay

*In one case this information was missing

Of 2805 patients with abdominal trauma in the trauma register (29% females), 243 patients (243/2805; 8.7%) had a renal injury of whom 182 underwent CT (182/243; 74.9%). The frequency of trauma CT protocols with regards to contrast phase, is presented in Table 3.

**Table 3:** Whole body trauma CT protocols

CT PROTOCOL/PHASE	*N*	(%)
Arterial and venous	66	36.3
Venous only	61	33.5
Venous and delayed nephrogram images	24	13.2
Arterial, venous and delayed nephrogram images	19	10.4
Arterial only	5	2.7
Arterial and delayed nephrogram images	2	1.1
Miscellaneous protocols	5	2.7
TOTAL	182	100%

Renal injury grading of the cohort according to AAST OIS 2018 revision is presented in Table 4

**Table 4:** Kidney injury grade according to AAST OIS 2018 revision. In 61 cases the information is either missing or was not assessed

GRADE	*N*	%
1	22	12.1
2	11	6.0
3	97	53.3
4	49	26.9
5	3	1.6
TOTAL	182	100

The most common injury mechanism was a high energy fall (31.7%), followed by motor vehicle traffic accidents (motorcycle and motor vehicle accidents) (28%) (Table 5)

**Table 5:** Injury mechanism in patients with kidney trauma

INJURY MECHANISM	*N*	%
High energy fall (fall from a higher level)	77	31.7
Traffic: motorcycle accident (the injured patient is an occupant or passenger of a motorcycle)	36	14.8
Traffic: motor vehicle accident – not motorcycle (the injured patient is an occupant or passenger of a motor vehicle; i.e., car, pickup truck, van, heavy transport vehicle, bus)	32	13.2
Stabbed by knife, sword, dagger, other pointed or sharp object	24	9.9
Struck or hit by blunt object (i.e., tree, tree branch, bar, stone, human body-part, metal, other)	22	9.1
Traffic: bicycle accident (the injured patient is an occupant or passenger of a bicycle)	22	9.1
Shot by handgun, shotgun, rifle, other firearm of any caliber	12	4.9
Low energy fall (fall at the same level)	8	3.3
Traffic: pedestrian (the injured patient is a pedestrian)	6	2.5
Traffic: other (the injured patient is an occupant or passenger of other means of transport; i.e., ship, airplane, railway train)	2	0.8
Unknown	2	0.8
	**243**	**100**

### Prevalence, injury pattern and management of renal vascular injury

Of the patients that underwent CT, 56 (56/182; 30.8%) had a renal vascular injury (Table 6).

**Table 6:** Patients with and without renal vascular injury (VI, no VI, respectively)

	VI (*n* = 56; 30.8%)	No VI (*n* = 126; 69.2%)	*p*
Gender (M/F)	48/8	98/28	0.2148
Age Side (L/R/bilat)	43.6 (15–78) 24/26/6	37.8 (15–83) 51/67/8	0.0409 0.6127
ISS	25 (4–57)	22 (4–75)	0.2584
NISS	29 (4–57)	27 (4–75)	0.2806
LOS days Injury (P/B)	14.9 (1-180) 11/45	14.7 (2-140) 10/116	0.9520 0.0416
Survived to discharge	52 (92.6%)*	120 (95.2%)	0.3165

P/B – Penetrating trauma/ blunt trauma

Survived to discharge = reciprocal of 30-day mortality = 30-day survival

*In one case this information was missing

Values are given in mean (age, LOS) or median (ISS, NISS). Range in parentheses

ISS, Injury Severity Score; NISS, New Injury Severity Score; LOS, hospital length of stay

These patients were slightly older than those not having a renal vascular injury (43.6 y vs. 37.8 y, *p* = 0.0409) and more often had a penetrating trauma (OR 2.836; 95% CI 1.127–7.136, *p* = 0.0416). No statistically significant difference was observed in injured side, ISS, NISS, LOS, or 30-day survival rate. The injury mechanism in patients with renal vascular trauma is presented in Table 7.

**Table 7:** Injury mechanism in patients with renal vascular trauma

Injury mechanism	*N*	%
High energy fall (fall from a higher level)	12	21.4
Stabbed by knife, sword, dagger, other pointed or sharp object	8	14.3
Traffic: bicycle accident (the injured patient is an occupant or passenger of a bicycle)	8	14.3
Struck or hit by blunt object (i.e., tree, tree branch, bar, stone, human body-part, metal, other)	7	12.5
Traffic: motor vehicle accident – not motorcycle (the injured patient is an occupant or passenger of a motor vehicle; i.e., car, pickup truck, van, heavy transport vehicle, bus)	7	12.5
Traffic: motorcycle accident (the injured patient is an occupant or passenger of a motorcycle)	6	10.7
Shot by handgun, shotgun, rifle, other firearm of any caliber	3	5.4
Low energy fall (fall at the same level)	2	3.6
Traffic: pedestrian (the injured patient is a pedestrian)	2	3.6
Traffic: other (the injured patient is an occupant or passenger of other means of transport; i.e., ship, airplane, railway train)	1	1.8
	**56**	**100**

Of the 56 patients with renal vascular injury, 42 (42/56; 75.0%) had active bleeding (two venous), 8 (8/56; 14.3%) a segmental infarct, 6 (6/56;10.7%) a PSA, and 3 (3/56; 5.4%) an arterial dissection (Table 8)

**Table 8:** Patients with renal vascular injury, *n* = 56

	Active bleeding (*n* = 42; 72.4%)	Segmental infarct (*n* = 8; 13.8%)	Pseudoaneurysm (*n* = 6; 10.3%)	Arterial Dissection (*n* = 3; 5.2%)	*p*
Gender (M/F)	36/6	7/1	6/0	2/1	0.5860
Age	47.6^†^ (16–78)	25.5^†^ (15–44)	42 (18–74)	36.7 (15–50)	*0.0191*
Side (L/R/Bilat)	18/22/2	3/3/2	1/4/1	2/0/1	0.5110
ISS	23.5 (4–57)	29 (16–57)	22 (9–34)	21 (17–48)	0.6267
NISS	29 (4–57)	33.5 (20–57)	29 (9–34)	24 (17–48)	0.7267
LOS days	16 (1-180)	11.8 (4–21)	12.5 (6–23)	12.3 (3–25)	0.7573
Injury (P/B)	9/33	0/8	4/2	0/3	0.0194^§^
Survived to discharge	38* (90.5%)	7 (87.5%)	6 (100%)	3 (100%)	0.7267

Three patients had both active bleeding and pseudoaneurysm

P/B – Penetrating trauma/ blunt trauma

Survived to discharge = reciprocal of 30-day mortality = 30-day survival

*In one case this information was missing

† denotes statistical difference between respective groups at *p* < 0.05 level

§ none of the pairwise comparisons remained statistically significant after follow-up pairwise comparisons using Dunn’s test

Values are given in mean (age, LOS) or median (ISS, NISS). Range in parentheses

ISS, Injury Severity Score; NISS, New Injury Severity Score; LOS, hospital length of stay

Three patients had both active bleeding and pseudoaneurysm. Patients with active bleeding were older than those with segmental infarct (*p* = 0.0191) and penetrating injury was most prevalent in patients with PSA (*p* = 0.0194). No statistically significant difference was seen in injured side, ISS, NISS, length of hospital stay or 30-day survival rate.

**Fig. 2:**
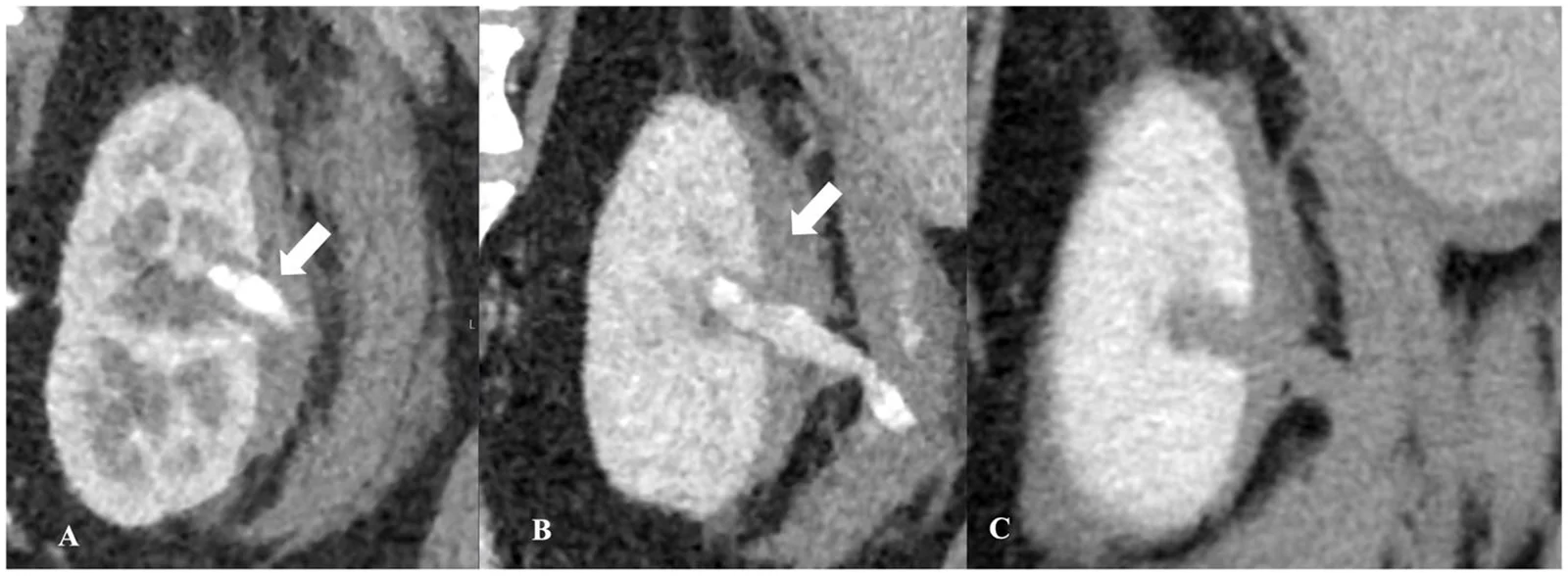
A 48-year-old male with a right-sided stab injury. Active arterial bleeding is seen on the arterial phase image (arrow, **A**), with an expanding hematoma on the venous phase image (arrow, **B**). The patient was managed non-operatively, with stable findings on the venous phase image obtained 24 h later (**C**)

Of the 42 patients with active bleeding, 31 (31/42; 73.8%) were managed non-operatively (Fig. 2).

**Fig. 3:**
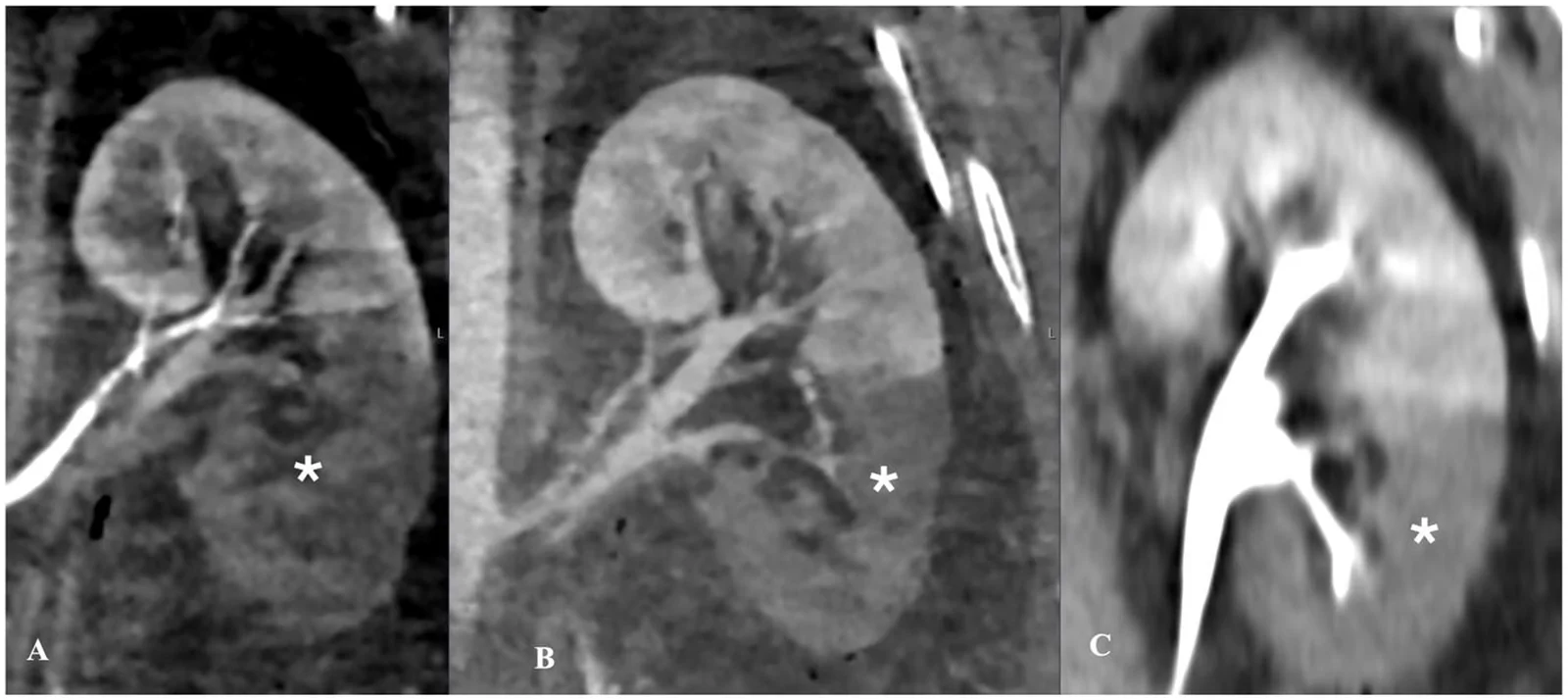
32-year-old male with a segmental infarct in the lower pole of the left kidney (asterisk). **A**) Arterial phase image. **B**) Venous phase image. **C**) Delayed phase image

Seven of these underwent an angiographic examination without subsequent intervention (coiling or stent etc.). Eight patients (8/42; 19.0%) underwent angioembolization, and three (3/42; 7.1%) underwent surgery. Six patients with segmental infarct were managed non-operatively (Fig. 3), and one underwent stent placement.

**Fig. 4:**
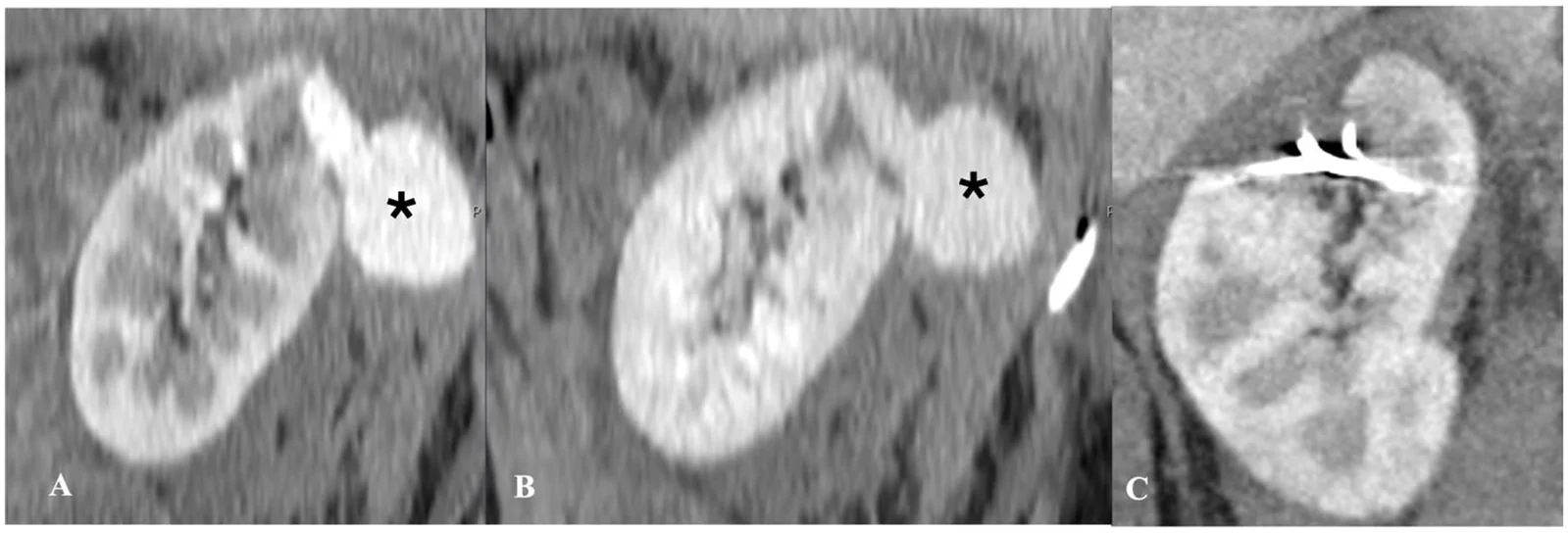
18-year-old male with a stab injury to the right kidney with a large PSA (asterisk). **A**) Arterial phase image. (**B**) Venous phase image. The PSA was treated with coil embolization (**C**)

For those having a PSA, three underwent angioembolization (Fig. 4), two were managed non-operatively, and one underwent surgery.

One of the three patients having both active bleeding and PSA underwent angioembolization. All patients with arterial dissection were treated non-operatively. Figure 1 shows renal injuries in patients with abdominal trauma at Karolinska University Hospital between 2013 and 2021.

### Concomitant injuries to liver, spleen, and kidney

Sixty-four patients had a concomitant splenic injury (26.3%; 64/243), 81 patients had a concomitant liver injury (33.3%; 81/243), and 18 patients had an injury to all three organs (7.4%;18/243), comprising 0.6% (18/2805) of all patients with abdominal trauma. CT was available for fourteen of them (Tables 9 and 10).

**Table 9:** Patients with injury to the liver, spleen and kidney (LSK)

	LSK (*n* = 14; 7.7%)	No LSK (*n* = 168; 92.3%)	*p*
Gender (M/F)	12/2	134/34	0.4086
Age	35.9 (16–57)	39.9 (15–83)	0.4709
ISS	34 (14–75)	22 (4–66)	*0.0047*
NISS	41 (16–75)	27 (4–75)	*0.0034*
LOS days	21.9 (3–45)	14.1 (1-180)	0.1755
Survived to discharge	13 (92.9%)	159 (94.6%)*	0.5237

Survived to discharge = reciprocal of 30-day mortality = 30-day survival

*In one case this information was missing

Values are given in mean (age, LOS) or median (ISS, NISS). Range in parentheses

ISS, Injury Severity Score; NISS, New Injury Severity Score; LOS, hospital length of stay

**Table 10:** Organ injury Grade in patients with concomitant injury to liver, spleen and kidney seen on admission CT (*n* = 14)

	LIVER	SPLEEN’	KIDNEY
G I	-	1	-
G II	7	4	1
G III	4	3	6
G IV	3	3	6
G V	-	2	1

*One patient had splenectomy before CT

The three-organ injury group had greater ISS and NISS, but no statistically significant difference was seen in LOS or in 30-day survival rate when compared to those not having an injury to all three organs

## Discussion

In our patient cohort, renal injuries were seen in 8.7% of patients with abdominal trauma. This is in line with some previously reported incidence rates [1]. Penetrating injury was the injury mechanism in 14.8% of patients, which is slightly higher than previously reported 9.2% [12] reflecting differences in local trauma profiles.

Arterial phase imaging is essential for detecting segmental or main renal artery occlusion, active bleeding as well as contained vascular injuries—findings that carry direct implications for management. The high sensitivity of MDCTA for detecting renal vascular injuries makes it the modality of choice in hemodynamically stable patients. In addition, delayed imaging that is performed 5 min post-injection, is needed if collecting system injury is suspected. In the current study, only half of the CT examinations had arterial phase, and only one fourth (24.6%) had delayed, excretory images. This demonstrates the importance of standardized CT protocols in trauma and polytrauma settings [13, 14].

Renal vascular injuries were not uncommon in our material. Of the patients who underwent CT on admission, almost one third (30.8%) had such an injury. One explanation for this might be our relatively high number of penetrating injuries (21/182; 11.5%), which are known to be associated with significantly higher rates of vascular involvement, particularly active bleeding. In the RVI group, 19.6% had a penetrating injury, and this number is almost identical to the frequency (19.5%) reported earlier by Alzerwi [12]. In contrast to a previously reported rate of 57.9% [12], traffic accidents (motorcycle and motor vehicle accidents) accounted for less than half (42.9%) of RVIs in our cohort. High-energy fall (21.4%) was the most common specific injury mechanism. This is plausible, as sudden deceleration is a recognized mechanism of injury in blunt renal trauma [15].

The management of renal trauma, including RVI, has evolved considerably over the past three decades, shifting from a predominantly operative approach toward selective non-operative management in hemodynamically stable patients [16–19]. Likewise, a majority of the patients in this study were also treated conservatively (Fig. 1), and almost three out of four (31/42) patients with active bleeding were managed non-operatively. Nevertheless, seven of these patients underwent angiography without subsequent intervention. The management of pseudoaneurysms varied, with three of six patients undergoing angioembolization, one undergoing surgery, and two managed conservatively. Although active bleeding and pseudoaneurysms are recognized indications for selective angioembolization, the European Association of Urology (EAU) guidelines state that validated criteria for selecting patients for angioembolization are lacking [2]. The small number of pseudoaneurysms in our cohort does not allow us to draw any conclusions regarding specific indications for intervention.

The EAU guidelines also recommend repeat imaging in patients with high-grade or penetrating renal injuries [2]. Furthermore, Barnes et al. [19] reported that some RVIs may resolve spontaneously on interval imaging, but new injuries, such as pseudoaneurysms can present up to 12 days after the initial injury [20]. However, the need for and optimal timing for in-hospital follow-up imaging were not specifically investigated in our study.

Concomitant injuries involving the kidneys, spleen, and liver, three highly vascularized abdominal organs, are serious and life-threatening conditions, requiring urgent multidisciplinary trauma care [21]. Fortunately, these injuries are relatively rare. Previously, we found in the same cohort that 2.3% of patients with abdominal trauma had an injury to both the spleen and liver [22]. In the current study, eighteen patients had an injury to the liver, spleen, and kidney, comprising 0.6% of all patients with abdominal trauma. No statistically significant difference was seen in LOS or in 30-day survival rate when compared to those not having an injury to all three organs.

In conclusion, RVI was not uncommon in our study cohort, occurring in 30.8% of patients for whom CT was available. Of these, 17.9% were treated with angioembolization. Patients with RVI were slightly older and were more likely to have a penetrating injury than patients without RVI. No statistically significant difference in hospital length of stay or 30-day survival was found between patients without and with RVI.

## Data Availability

The data supporting the findings of this study are available from the corresponding author upon reasonable request.
